# Interferon Gamma in African Trypanosome Infections: Friends or Foes?

**DOI:** 10.3389/fimmu.2017.01105

**Published:** 2017-09-07

**Authors:** Hui Wu, Gongguan Liu, Meiqing Shi

**Affiliations:** ^1^Department of Obstetrics and Gynecology, Nanjing First Hospital, Nanjing Medical University, Nanjing, China; ^2^Division of Immunology, Virginia-Maryland Regional College of Veterinary Medicine, University of Maryland, College Park, MD, United States

**Keywords:** *Trypanosoma brucei*, *Trypanosoma congolense*, interferon gamma, protection, immunopathology

## Abstract

African trypanosomes cause fatal infections in both humans and livestock. Interferon gamma (IFN-γ) plays an essential role in resistance to African trypanosomes. However, increasing evidence suggests that IFN-γ, when excessively synthesized, also induces immunopathology, enhancing susceptibility to the infection. Thus, production of IFN-γ must be tightly regulated during infections with African trypanosomes to ensure that a robust immune response is elicited without tissue destruction. Early studies have shown that secretion of IFN-γ is downregulated by interleukin 10 (IL-10). More recently, IL-27 has been identified as a negative regulator of IFN-γ production during African trypanosome infections. In this review, we discuss the current state of our understanding of the role of IFN-γ in African trypanosome infections. We have focused on the cellular source of IFN-γ, its beneficial and detrimental effects, and mechanisms involved in regulation of its production, highlighting some recent advances and offering some perspectives on future directions.

## Introduction

African trypanosomiasis is a parasitic disease of humans and animals (1, 2). The disease is fatal if left untreated and mainly found in sub-Saharan Africa. In the past years, much effort has been made to confront the disease, and the reported cases are declining. However, there are still about 17,000 cases of infections in humans and approximately 70 million people are at the risk of contracting the disease (3, 4). In addition, an estimated three million cattle die from this disease each year, resulting in economic loss of four billion US dollars in Africa (5). Following malaria and schistosomiasis, African trypanosomiasis is the third significant contributor to the global burden of parasitic diseases (6). The causative agents of this disease are various species of protozoan parasites belonging to genus of *Trypanosoma*. Among them, *Trypanosoma brucei rhodesiense* and *Trypanosoma brucei gambiense* infect humans, while *Trypanosoma brucei brucei, Trypanosoma congolense*, and *Trypanosoma vivax* are the major species causing animal infections (6).

**Figure 1:**
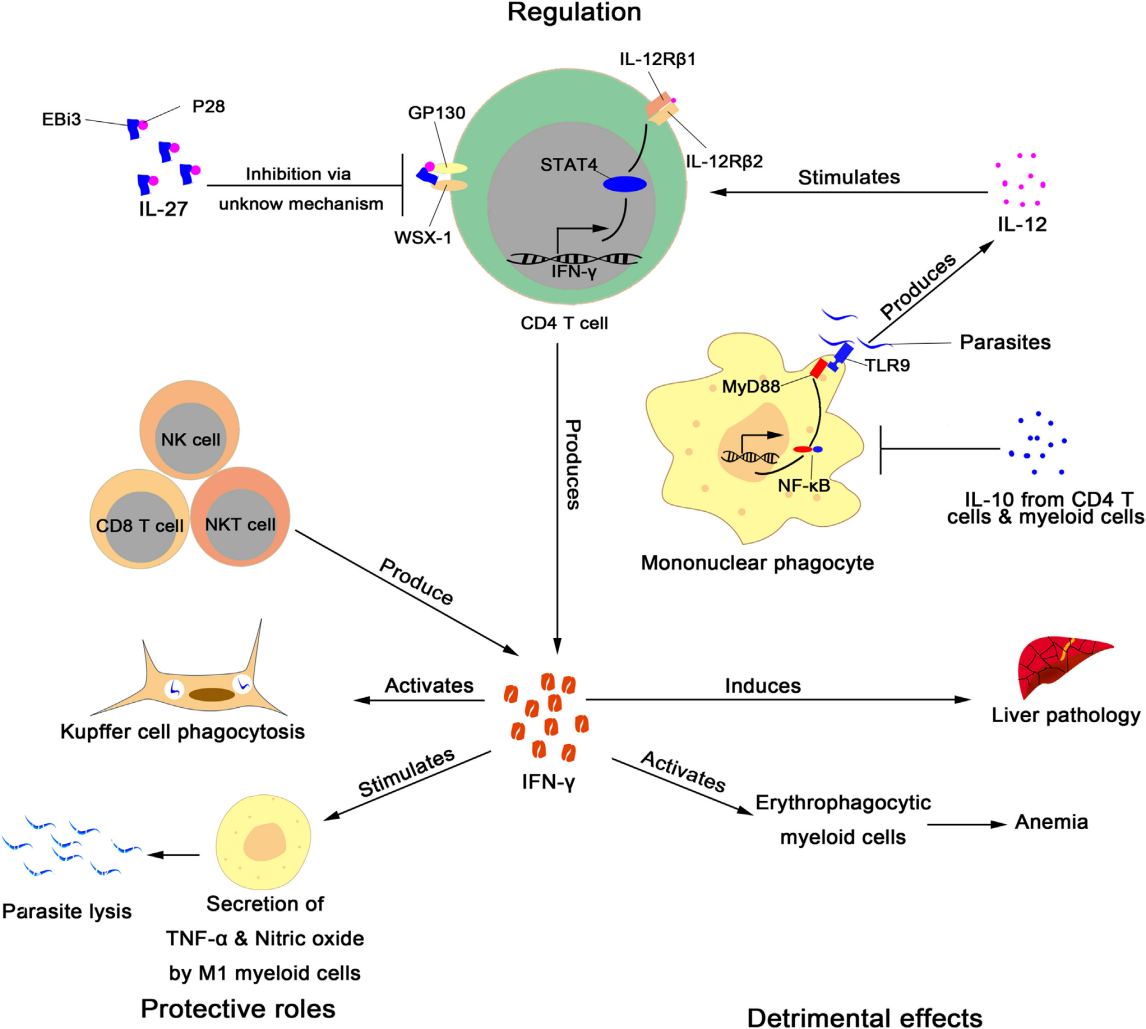
Role and regulation of interferon gamma (IFN-γ) during infection with African trypanosomes. Regulation: CD4^+^ T cells (16, 18, 21–24), CD8^+^ T cells (18–20), NK cells (18), and NKT cells (18) produce IFN-γ during infection with African trypanosomes. African trypanosomes activate mononuclear phagocytes to secrete IL-12 through TLR9 and MyD88 signaling (25). IL-12 stimulates CD4^+^ T cells to produce IFN-γ *via* activation of STAT4 signaling (26), whereas IL-27 inhibits CD4^+^ T cells to secrete IFN-γ (24). Interleukin 10, mainly synthesized by CD4^+^ (22, 23, 27) and myeloid cells (28), inhibits IFN-γ production through downregulation of the secretion of IL-12 by direct modulation of mononuclear phagocytes (2). Protective role: IFN-γ enhances Kupffer cell phagocytosis of trypanosomes circulating in the bloodstream (12, 13). IFN-γ also promotes M1 myeloid cells to produce TNF-α and nitric oxide, which mediate parasite lysis (16, 29–31). Detrimental effects: excessive secretions of IFN-γ lead to liver pathology (12, 24, 26, 32) and activation of erythrophagocytic myeloid cells, resulting in anemia (18).

African trypanosomiasis is transmitted by tsetse fly. After tsetse fly takes a blood meal from infected mammalian hosts, African trypanosomes multiply in tsetse fly’s midgut and migrate to the salivary glands. Upon the bite of an infected tsetse fly, the parasites are inoculated into the mammalian host, and replicate in the bloodstream and interstitial fluids of the mammalian host, and at a later stage, they can invade the brain and cause fatal meningoencephalitis (3, 4). As extracellular pathogens, the organisms are directly exposed to immune cells circulating in the bloodstream (2). To survive, the parasites have evolved very sophisticated mechanisms, including antigenic variation of the variant surface glycoprotein, to evade the host immune responses (2). Mouse models have been widely used to study the host immune responses to African trypanosomes (7). Based on mouse models, the liver is the major place for clearance of the parasites circulating in the bloodstream (8–10); Kupffer cells, residing within the lumen of the liver sinusoids, play a prominent role in the phagocytosis of the parasites in an IgM- and IgG-dependent manner (11–14). Phagocytosis of African trypanosomes is associated with an outburst of production of cytokines, which are involved not only in resistance but also susceptibility of mice to the parasite (2). Among these, interferon gamma (IFN-γ) has emerged as an important cytokine dictating the disease outcome during African trypanosomiasis. Early studies demonstrated that IFN-γ is essential for resistance to African trypanosomes (15, 16). However, more recent results suggest that IFN-γ, when excessively secreted, mediates susceptibility to trypanosome infections (12, 17). Below, we review the cellular source of IFN-γ during infections with African trypanosomes (Table 1), discuss the beneficial and detrimental effects of IFN-γ, and explore the molecular mechanisms that regulate production of this cytokine (Figure 1).

**Table 1:** Cellular source of interferon gamma during infection with African trypanosomes.

Subsets of leukocytes	Mouse strains	Trypanosome strains	Approaches for detection	Reference
NK cells	C57BL/6	*Trypanosoma brucei brucei* AnTat1.1E	Flow cytometry	Cnops et al. (18)
NKT cells	C57BL/6	*T. brucei brucei* AnTat1.1E	Flow cytometry	Cnops et al. (18)
CD8^+^ T cells	DBA/2	*T. brucei brucei* AnTat1.1E	Knockout mice; immunospot	Olsson et al. (19)
	C57BL/6	*T. brucei brucei* AnTat1.1E	Knockout mice; ELISA	Namangala et al. (20)
	C57BL/6	*T. brucei brucei* AnTat1.1E	Flow cytometry	Cnops et al. (18)
CD4^+^ T cells	B10.BR	*Trypanosoma brucei rhodesiense* LouTat 1	Flow cytometry, ELISA	Schleifer et al. (21)
	BALB/c	*Trypanosoma congolense* TC13	Knockout mice, flow cytometry, immunocytochemistry, ELISA	Shi et al. (22)
	C57BL/6	*T. congolense* TC13	Knockout mice, ELISA	Magez et al. (16)
	BALB/c	*T. brucei brucei* 10-26	Knockout mice, ELISA	Liu et al. (23)
	C57BL/6	*T. congolense* TC13	CD4 depletion, flow cytometry, ELISA	Liu et al. (24)
	C57BL/6	*T. brucei brucei* AnTat1.1E	Flow cytometry	Cnops et al. (18)

## Cellular Source of IFN-γ

Interferon gamma was discovered in 1965 (33). IFN-γ is a protein with 146 amino acids residues, the sole member of the type 2 interferon family, and mediates most of the cell responses through JAK–STAT pathway (34). As a major regulator of immune responses, IFN-γ is produced by multiple types of immune cells. At the early stage of infections by many pathogens, IFN-γ is rapidly secreted by NK cells and NKT cells (35, 36). In general, CD4^+^ type 1T helper cells and CD8^+^ cytotoxic T cells are the major producer of IFN-γ during infections (37). In addition, γδ T cells and some myeloid cells have also been reported to have the potential to secrete IFN-γ (37–41).

During infection with *T. brucei rhodesiense* in a mouse model, a subset of VSG-specific TCRαβ^+^ CD4^+^, but CD8^−^, T cells has been shown to secrete high levels of IFN-γ (21). Similar to infection with *T. brucei rhodesiense*, IFN-γ is produced predominantly by CD3^+^Thy1.2^+^TCRαβ^+^CD4^+^ T cells in BALB/c mice infected with *T. congolense*, as demonstrated by both immunohistochemistry and flow cytometry (22). Accordingly, secretion of IFN-γ was almost abolished in CD4^−/−^ BALB/c mice infected with *T. congolense* and infected mice depleted of CD4^+^ T cells (22). Analysis of IFN-γ secretion by spleen cells from *T. congolense*-infected MHC-II^−/−^ C57BL/6 mice also revealed a complete abrogation of IFN-γ production (16). These results demonstrate that IFN-γ is mainly produced by CD4^+^ T cells during infection with African trypanosomes.

In contrast, it was also reported that *T. brucei brucei* AnTat1.1E produces a T lymphocyte-triggering factor, which stimulates CD8^+^ T cells to secrete IFN-γ (19, 42). CD8^+^ T cell released IFN-γ, as a growth-stimulating factor, promotes parasite growth (19, 42). Neutralization of IFN-γ suppressed parasite growth and increased the survival of mice infected with *T. brucei brucei* AnTat1.1E (43). In support of these data, CD8^−/−^ C57BL/6 mice infected with *T. brucei brucei* AnTat1.1E had lower parasitemia and survived significantly longer compared to infected wild-type mice (19). These data suggest that CD8^+^ T cells kill mice *via* their secretions of IFN-γ during *T. brucei brucei* infections. The detrimental role of CD8^+^ T cells has been recently confirmed in BALB/c mice, as CD8^−/−^ mice in BALB/c background also survived significantly longer than the wild-type cohorts following infection with *T. brucei brucei* 10-26 (23). Surprisingly, IFN-γ production was completely abrogated in CD4^−/−^, but not CD8^−/−^ BALB/c mice infected with *T. brucei brucei* 10-26 based on measurement of IFN-γ in plasma and spleen cell cultures, suggesting that IFN-γ is produced mainly by CD4^+^ T cells, but not CD8^+^ T cells in BALB/c mice infected with *T. brucei brucei* 10-26 (23), which is consistent to the observation in mice infected with *T. congolense* (16, 22). Thus, the cellular source of IFN-γ during infections with African trypanosomes is controversial. One possible explanation for these discrepancies may lie with the strains of infected mice, the species and strains of parasites, and the stages of infections. Indeed, it has been more recently reported that NK and NKT cells are the earliest producer of IFN-γ in C57BL6 mice during *T. brucei brucei* AnTat1.1E infection, and that later in infection, IFN-γ is mainly produced by CD8^+^ and CD4^+^ T cells (18), supporting the notion that IFN-γ is produced by different subsets of leukocytes at different stages of infections.

## Protective Role of IFN-γ

Interferon gamma plays essential roles in protective immunity against infections, particularly infections with viruses and intracellular bacteria and protozoan parasites (35, 36). IFN-γ is involved in Th1 differentiation, activation of antigen-presenting cells, secretions of pro-inflammatory cytokines, and leukocyte migration to the site of infection (35, 36). Early studies have shown a correlation between high IFN-γ levels in serum, low levels of parasitemia, and host resistance in mice infected with *T. brucei rhodesiense* (44). IFN-γ^−/−^ C57BL/6 mice infected with *T. brucei rhodesiense* could control the first wave of the parasitemia; however, they displayed higher parasitemia than infected wild-type control (15). Following infection with *T. brucei rhodesiense*, IFN-γ^−/−^ C57BL/6 mice survived only 19 days, while infected wild-type cohorts were found to survive an average of 46 days; passive transfer of spleen cells from wild-type mice to IFN-γ^−/−^ mice significantly enhanced the survival, demonstrating that IFN-γ is critical for host resistance (15). Similar to infection with *T. brucei rhodesiense*, IFN-γ^−/−^ C57BL/6 mice infected with *T. brucei brucei* also survived significantly shorter compared to infected wild-type mice (20). In support of the protective role of IFN-γ, IFN-γR^−/−^ C57BL/6 mice infected with *T. congolense* failed to control the first wave of parasitemia and succumbed to infection on day 24 postinfection, while infected wild-type control could survive more than 100 days postinfection (16).

Interestingly, MHC II^−/−^ C57BL/mice infected with *T. congolense*, which did not produce IFN-γ, were unable to control the first wave of parasitemia (16) and survived significantly shorter than infected wild-type mice (16, 45). Furthermore, MyD88^−/−^ and TLR9^−/−^ C57BL/6 mice produced significantly less IFN-γ following infection with *T. brucei brucei*, coinciding with impaired parasite clearance and reduced survival (25). Similarly, the secretion of IFN-γ was severely impaired in IL-12p70^−/−^ C57BL/6 mice infected with *T. brucei brucei* or *T. evansi*, which was associated with significant reduction of survival time (46). Since IFN-γ^−/−^ or IFN-γR^−/−^ mice are more susceptible to infection with African trypanosomes than wild-type mice (15, 16), it is conceivable that the early mortality of infected MyD88^−/−^, TLR9^−/−^, and IL-12p70^−/−^ mice is attributed to, at least in part, the impaired capacity of synthesis of IFN-γ (25, 46).

Importantly, a recent report demonstrated that IFN-γ was only detected in the whole blood derived from “trypanotolerant” patients with latent *T. brucei gambiense* infections, but not patients with active disease, following incubation with the parasites *in vitro* (47). This result suggests that IFN-γ production may be also linked to resistance to African trypanosomes in clinical settings (47).

Although it is known that IFN-γ is required for resistance to African trypanosomes, the underlying mechanism(s) remains poorly understood. As mentioned above, Kupffer cells in the liver play an important role in the clearance of circulating parasites (12, 13); it is possible that IFN-γ promotes the activation of Kupffer cells, enhancing the efficiency of trypanosome phagocytosis by Kupffer cells. It is also likely that IFN-γ exerts its protective effect through stimulation of M1-type myeloid cells, resulting in secretion of tumor necrosis factor alpha (TNF-α) and nitric oxide, which are known to mediate parasite lysis or death (16, 29–31).

## Detrimental Effects of IFN-γ

Animal evidence suggests that there is considerable genetic variation in susceptibility to African trypanosomiasis. For example, the Zebu breed of cattle is more susceptible than the indigenous West African N’dama breed to infections with *T. congolense* (48). In laboratory models, BALB/c mice are highly susceptible to African trypanosomes and can only survive 7–10 days following *T. congolense* infections, while C57BL/6 are relative resistant and can survive >100 days (2, 49). Although the mechanisms underlying the differences in susceptibility are poorly understood, it was reported that there is dramatic difference in the cytokine profiles between infected BALB/c and C57BL/6 mice (49). In particular, plasma levels of IFN-γ are significantly higher in susceptible BALB/c mice infected with *T. congolense* compared to infected C57BL/6 mice (17, 50). Interestingly, *in vivo* administration of anti-IFN-γ antibodies, early during infection with *T. congolense* reduced parasitemia and dramatically prolonged the survival time of BALB/c mice, suggesting that IFN-γ has deleterious effects and contributes to the relative susceptibility of BALB/c mice to the disease (17).

In contrast to the fact that trypanosome-susceptible BALB/c mice can be altered to a relatively resistant-like phenotype by neutralization of IFN-γ (17), enhancing the production of IFN-γ by blocking IL-10R during *T. congolense* or *T. brucei brucei* infections can also switch the resistant C57BL/6 mice to a susceptible-like phenotype (12). Infected C57BL/6 mice treated with anti-IL-10R displayed significantly higher plasma levels of IFN-γ than the control group, which was associated with an early mortality (died on 7–10 days post infection); the early death of the infected mice could be prevented by neutralization of IFN-γ, demonstrating that IFN-γ is the killer (12). Infected mice treated with anti-IL-10R exhibited increased plasma levels of proinflammatory cytokines and extensive focal necrosis in the liver, suggesting that IFN-γ mediates liver pathology (12). IFN-γ mediated liver injury was further confirmed using IL-12p70^−/−^ C57BL/6 mice, as IL-12p70^−/−^ mice showed strikingly reduced IFN-γ production, coinciding with a dramatic drop of plasma aspartate transaminase and a significant enhanced survival during infection with *T. congolense* (26).

In addition to liver pathology, more recent results have demonstrated that IFN-γ also accounts for acute anemia, one of the major characteristics of African trypanosomiasis (18). Acute anemia is caused by enhanced erythrophagocytosis mediated by activated cells of the myeloid phagocytic system in trypanosomiasis (51). IFN-γ has been shown to be critically involved in the recruitment and activation of erythrophagocytic myeloid cells; as such IFN-γR^−/−^ mice were partially protected against trypanosomiasis-associated inflammation and acute anemia, demonstrating a detrimental role of IFN-γ in driving enhanced erythrophagocytosis by myeloid phagocytic cells and the induction of acute inflammation-associated anemia (51).

Why does IFN-γ have both protective and detrimental effects during infections with African trypanosomes? It may depend on how much IFN-γ is produced during the infection. It turns out that IFN-γ is protective; however, excessive production of this cytokine may worsen the disease. This notion was supported by an experiment performed using BALB/c mice infected with *T. congolense* (22). As CD4^+^ T cells are the major producer of IFN-γ during *T. congolense* infection (16, 22, 23) and excessive production of IFN-γ kills infected BALB/c mice (17), a strategy was developed with the aim to maintain an optimal production of IFN-γ by partial depletion of CD4^+^ T cells using high and low doses of ant-CD4 mAb (22). Strikingly, infected BALB/c mice partially (0.1 mg mAb), but not completely (4 mg mAb), depleted of CD4^+^ T cells had significantly decreased parasitemia and dramatically enhanced survival, accompanied with ~80% reduced production of IFN-γ (22). By contrast, mice without depletion of CD4^+^ T cells (high plasma levels of IFN-γ) or mice depleted completely of CD4^+^ T cells (undetectable plasma level of IFN-γ) were unable to control the first wave of parasitemia and died on day 7–10 postinfection (22). These results strongly suggest that an optimal, but not excessive, amount of IFN-γ is essential for host resistance to African trypanosomes without causing severe immunopathology.

## Regulation of IFN-γ

As discussed above, IFN-γ plays an essential role in host defense against African trypanosomes. It has been reported that MyD88^−/−^ and TLR9^−/−^ mice had significantly decreased plasma levels of IFN-γ during *T. brucei brucei* infection, suggesting that these two signals positively regulate secretions of IFN-γ (25). In addition, IL-12p70^−/−^ mice infected with *T. congolense* exhibited lower plasma levels of IFN-γ compared to infected wild-type mice, demonstrating that IL-12 promotes IFN-γ secretions (26). However, uncontrolled IFN-γ production is also detrimental to the host. Thus, IFN-γ production must be tightly regulated.

Interleukin 10 (IL-10) is an immunoregulatory cytokine with primary function in limiting inflammation. Studies from mouse models have shown that IL-10 is essential for maintenance of the immunological balance between protective and pathological immune responses during African trypanosomiasis (12, 20, 32, 52, 53). The anti-inflammatory function of IL-10 has been also confirmed in cattle, primate, and human infections with African trypanosomes (54–56). Suppression of IFN-γ by IL-10 was firstly reported by Namangala et al. (20) who found that IL-10^−/−^ mice infected with *T. brucei brucei* had significantly higher amounts of plasma IFN-γ and died earlier compared to infected wild-type mice (20). Accordingly, blocking IL-10R also significantly enhanced the plasma levels of IFN-γ, leading to early death of C57BL/6 mice during *T. congolense* infection (12). Interestingly, IL-10 production was significantly impaired in CD4^−/−^ mice infected with *T. brucei brucei* (20, 23) and *T. congolense* (22), suggesting that IL-10 is secreted at least partially by CD4^+^ T cells. In support of this, Foxp3^+^ CD4^+^ Tregs have been found to expand in the spleen and the liver of mice infected with *T. congolense*, limiting IFN-γ production *via* their secretions of IL-10 (27). In addition, recent data suggest that Ly6C^−^ monocytes and macrophages are also the cellular source of IFN-γ during *T. congolense*-infections (28). Collectively, IL-10, mainly secreted by CD4^+^ and myeloid cells, negatively regulates IFN-γ production. It is likely that IL-10 inhibits IFN-γ production through downregulation of the secretion of IL-12 and TNF-α by direct modulation of M1-type myeloid cells.

IL-27, a recently identified cytokine produced primarily by macrophages and dendritic cells, has been shown to downregulate inflammation (57). A recent study has shown that IL-27R^−/−^ mice infected with *T. congolense* or *T. brucei brucei* developed severe liver pathology and showed a shorter survival, coinciding with overactivation of CD4^+^ T cells and excessive production of IFN-γ (24). Neutralization of IFN-γ or depletion of CD4^+^ T cells prevented the early mortality of infected IL-27R^−/−^ mice (24). Interestingly, IL-10 production was not impaired in infected IL-27R^−/−^ mice (24). These results demonstrate that IL-27 inhibits IFN-γ secretion by CD4^+^ T cells in an IL-10-independent manner, preventing immunopathology during African trypanosome infections.

## Concluding Remarks

Interferon gamma is critically involved in immunomodulation in infectious diseases. The cellular source and role of IFN-γ are controversial during infections with African trypanosomes. Although antigen-specific CD4^+^ T cells have been shown to be the major producer of IFN-γ (16, 21–23), recent results suggest that other subsets of immune cells including NK cell, NKT cell, and CD8 T cells also contribute to the secretions of IFN-γ, in particular, at the early stage of infections (18). IFN-γ is required for resistance to African trypanosomes (15, 16, 20), but when excessively secreted, it also mediates immunopathology (12, 17, 24). Thus, IFN-γ is a double edged sword and its production must be tightly controlled. IL-10 and IL-27 play essential roles in negative regulations of IFN-γ secretions *via* different mechanisms (12, 20, 24). It still remains poorly understood how IFN-γ exerts its detrimental effect. In this regard, recent work demonstrates that TNF-α and iNOS producing dendritic cells (Tip-DCs) also play a deleterious role during African trypanosomiasis (32, 53). Therefore, future work on this issue will likely be focused on determination of whether a link among IFN-γ, Tip-DCs, and IL-27 exists.

## Author Contributions

All authors listed have made a substantial, direct, and intellectual contribution to the work and approved it for publication.

## Conflict of Interest Statement

The authors declare that the research was conducted in the absence of any commercial or financial relationships that could be construed as a potential conflict of interest.
